# Calcium Signaling and Molecular Adhesion Processes May Hold the Key to Genetic Risk for Autism: A Molecular Pathway Analysis on Two Independent Samples

**DOI:** 10.3390/genes15121609

**Published:** 2024-12-17

**Authors:** Antonio Drago, Marco Calabro, Concetta Crisafulli

**Affiliations:** 1 Department of Clinical Medicine, Aalborg University, 9220 Aalborg, Denmark; 2 Department of Biomedical Science and Morphological and Functional Images, University of Messina, Via Consolare Valeria, 98125 Messina, Italy; calabro.marco@outlook.it (M.C.); concetta.crisafulli@unime.it (C.C.)

**Keywords:** autism, gene, molecular pathway analysis, SNP

## Abstract

Background: Autism spectrum disorder (ASD) is a neurodevelopmental disorder characterized by limited interests, difficulties in social interactions, repetitive behaviors, and impairments in social communication. ASD tends to run in families, and twin studies suggest a strong genetic basis for the disorder. However, the definition of a genetic profile that indicates a risk for ASD remains unclear. Methods: This analysis includes an investigation (Autism Dataset 4 from the NIMH repository, n = 2890) and a replication (Autism Dataset 3 from the NIMH repository, n = 1233) of trio samples with GWAS data. In Phase 1, a molecular pathway analysis is conducted on the investigation sample to test for the enrichment of specific Gene Ontology (GO) terms associated with autism. In Phase 2, the identified pathways are tested for enrichment in the replication sample. Permutation tests are performed to reduce the risk of false-positive findings. Quality assessment is conducted using QQ-plots and λ values, with Plink and R utilized for the Transmission Disequilibrium Test (TDT) and permutation tests. Results: The GO term GO:0007417 was found to be enriched in both the investigation and replication samples. SNPs associated with this pathway were observed at a frequency higher than expected in the replication sample. Conclusions: The GO term GO:0007417 (development of the nervous system) was associated with autism in both trio samples. Variations in the genes *TMPRSS4*, *TRPC4*, and *PCDH9* were consistently linked to autism across the two independent samples, highlighting the role of calcium signaling and cell adhesion molecules in the risk of autism-related disorders. The pathways and variations associated with autism are described in detail, which can contribute to the engineering of new pharmacological treatments for ASD.

## 1. Introduction

Autism spectrum disorder (ASD) is a neurodevelopmental disorder that manifests early in life. It is characterized by limited interests, difficulties in social interactions, repetitive behaviors, and impairments in social communication. Additionally, individuals with ASD often experience motor and intellectual deficits, as well as mood and sleep disorders, along with sensory and gastrointestinal abnormalities [1]. ASD affects up to 1% of the general population [2] and has a genetic component, with an estimated concordance rate of 60–70% in identical twins and 5–30% in siblings [3,4].

In May 2014, the sixty-seventh World Health Assembly adopted a resolution entitled comprehensive and coordinated efforts for the management of autism spectrum disorders, which was supported by more than 60 countries. ASD is associated with approximately 3.6 million USD in lifetime social costs [5].

A recent review on the molecular aspects of ASD can be found in [6].

The GWAS Atlas (https://atlas.ctglab.nl/) accessed 15 July 2024 reports on two large GWAS focused on autism [7,8]. In the first study [7], including 7387 cases and 8567 controls, only one SNP reached GWAS significance, rs7026354, a G/A variation with no previous recorded clinical significance and which is not associated with a specific gene. In the second study, which included 18,381 cases and 27,969 controls, three SNPs reached GWAS significance: rs10099100, rs71190156, and rs910805 [8]. Rs10099100 G > C SNP is located in chr 8, and it is not reported to have clinical significance, rs71190156 is a *MACROD2*, intron variant, and rs910805 is located on chr 20. None of them have known clinical significance.

Environmental factors that may increase the risk of ASD are estimated to account for 50% of the risk and can lead to epigenetic modifications of key genes [9]. Epigenetic modifications are not only involved in the biological processes associated with the gene–environment interactions but also play a crucial role in orchestrating the various protein and genetic events that drive neurodevelopment [10]. Consistently, ASD is thought to be a neurodevelopmental disorder. A number of genetic variations have been inconsistently associated with an increased risk of ASD [9], and GWAS investigations have not consistently identified the single critical variations associated with this disorder [11]. This contrasts with evidence indicating that the heritability of ASD due to common variants is estimated to be as high as 80%, as recently reviewed in [12]. Limited power of the analysis, small sample sizes, and a critical phenotype definition may be limitations of the studies conducted so far [11]. Moreover, de novo mutations may explain a part of the missing heritability, as it was consistently shown that they may play a role in ASD [13,14,15,16]. Nevertheless, the impact of de novo mutations is less significant than that of inherited variants. It is estimated that 49% of the genetic architecture of ASD is associated with common inherited variants, while de novo mutations account for 3%, and rare inherited variants also account for 3% [17]. De novo mutations can lead to syndromes that resemble ASD, differing primarily in certain epidemiological details, such as a higher incidence of dysmorphic features and a similar prevalence between males and females. Examples of such genetic conditions include Fragile X syndrome, Tuberous Sclerosis Complex, Dup15q syndrome, deletions in the 16p11.2 region, Rett syndrome, and neurofibromatosis. These conditions are estimated to account for up to 5% of ASD cases observed in clinical practice [18]. There is currently no conclusive evidence for a limited number of critical common variations that confer risk for most cases of ASD. However, it is estimated that an additive effect of single variations, each having a small impact on the phenotype, may explain 60% of the genetic liability in multiplex families and 40% in simplex families [19]. An updated list of the genetic variations and genes associated with ASD can be downloaded at https://gene.sfari.org/autdb/GS_Home.do (accessed 15 July 2024). The number of genes and variations associated with ASD is extensive, and the replication rate for individual genes or mutations does not support conclusive conclusions about the neuropathology of the disorder. A potential solution to this sparse genetic evidence is the “many genes, common pathways” hypothesis, which posits that the effects of different genes may converge on common pathways and that impaired function in these pathways results in the core symptoms of ASD. Analyzing molecular pathways can enhance our understanding of complex genetic diseases by identifying gene sets that exhibit an enriched profile of association with the phenotype in question. This approach aligns with the polygenic nature of complex disorders and considers the cumulative small effects of individual genetic variations on complex phenotypes, as seen in [20,21]. Previous investigations into genetic networks which disruption could lead to ASD have identified several molecular pathways that may be involved, including protein synthesis and metabolism, the modulation of transcription processes, chromatin remodeling, calcium signaling, and the oxytocin pathway [10,22]. Nevertheless, those findings are generated by systematic reviews, and to the best of our knowledge, a molecular pathway analysis on GWAS data derived from ASD trios has yet to be conducted. It must be noted that the following investigation is conducted on the information derived from the distribution of SNPs (single nucleotide polymorphisms). SNPs are probably the most prevalent form of genetic variation in the human genome (3 to 4 million). Consequently, SNPs serve as an extensive and valuable source of information [23].

Molecular pathway analysis may be a promising approach to GWAS data, but it also has limits. One of the main limits of the genome-wide molecular pathway analysis is that it relies on known molecular pathways and known genetic functions [24]. Moreover, this technique is limited by the number of available SNPs included in GWAS, which means that the risk of false-negative findings due to the inadequate coverage of specific genes cannot be ruled out. Moreover, European and North American populations are overrepresented in the international populations. There is a clear need for non-European and North American samples to be investigated. With this in mind, it is possible to combine the results of molecular pathway analysis with the current published evidence regarding specific phenotypes, thereby helping to define the genetic makeup that increases the risk for a disease or group of diseases. In the present investigation, a molecular pathway analysis is applied to two available GWAS databases on autism disorder (investigation and replication samples). The genes harboring variations significantly associated with autism in one sample are tested for their involvement in specific molecular pathways associated with the disorder in both samples. As a result of this investigation, the identification of a molecular pathway associated with increased risk for autism is expected.

## 2. Methods

### 2.1. Dataset

Genetic data were available from NIMH genetics (https://www.nimhgenetics.org/) accessed 15 July 2024. The Autism Dataset 4 (Study 65/TASC GWAS Data) sample was chosen for the investigation sample. The Autism Dataset 3 (GWAS Data on 1264 Non-AGRE Samples) served as a replication sample. GWAS were conducted with Affymetrix 5.0 in both samples.

### 2.2. Toolset

Plink [25] served for the TDI GWAS analysis and genetic annotations. Plink was chosen because it is the open-source gold standard for GWAS analysis. R [26] and dedicated packages served for the permutations analysis and QQ-plot creation. R was chosen along with dedicated packages because it is an open-source widely used platform for statistical analysis. Haploview [27] served for the identification of the known available SNPs for each gene. The Gene Ontology Consortium (http://geneontology.org/) accessed 15 July 2024 was interrogated to detect enriched GO annotations. Given a list of genes, the Gene Ontology Consortium will provide a list of GO terms that identify the molecular pathways in which the genes are embedded. This process is made possible by the implementation of the R packages that were used for the analysis. Annotations of single variations were manually curated, and the source of information was an international database on genetic variations (www.gwascentral.org, accessed on 15 July 2024). This information is also accessed through the R dedicated packages. The enrichment analysis was conducted using the R software (R-4.3) suite through Bioconductor [28] and the package ReactomePA [29]. Reactome [30] is a manually curated database the includes chemical reactions, biological processes, and molecular pathways. Further investigations on elements belonging to the enriched pathways were performed through Cytoscape software 3.10 [31] and the plugins String 1.5.1, GeneMania 1.0, ClueGO 0.3-66, and CluePedia 0.3-66. Here, the physical and genetic interactions between the elements were investigated to produce an interaction network between the six enriched pathways. Further, the same approach was used to extend the network to include drugs interactions (using the Cytargetlinker 4.1.0 plugin and Drugbank Database (ver. 5.6.1)).

**Chart 1:**
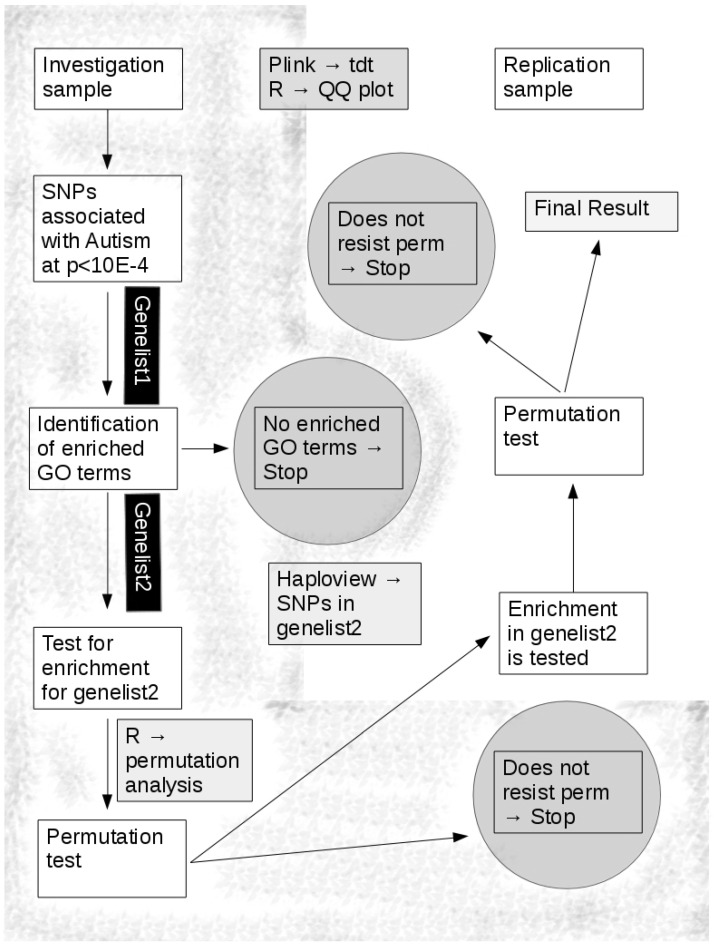
The analysis flow is shown. The shadowed areas refer to the analysis of the investigation sample. Circles indicate the points in which the analysis is meant to stop, and a negative result is given. Arrows show the direction of the analysis. Enrichment in genelist2 in the replication sample is conducted while accounting for *p* thresholds < 0.01 and a consistent direction of association between the investigation and the replication sample (OR> or <1).

Chart 1 shows the protocol of the analysis.

### 2.3. Analysis Flow

#### 2.3.1. Investigation Sample

**Figure 1:**
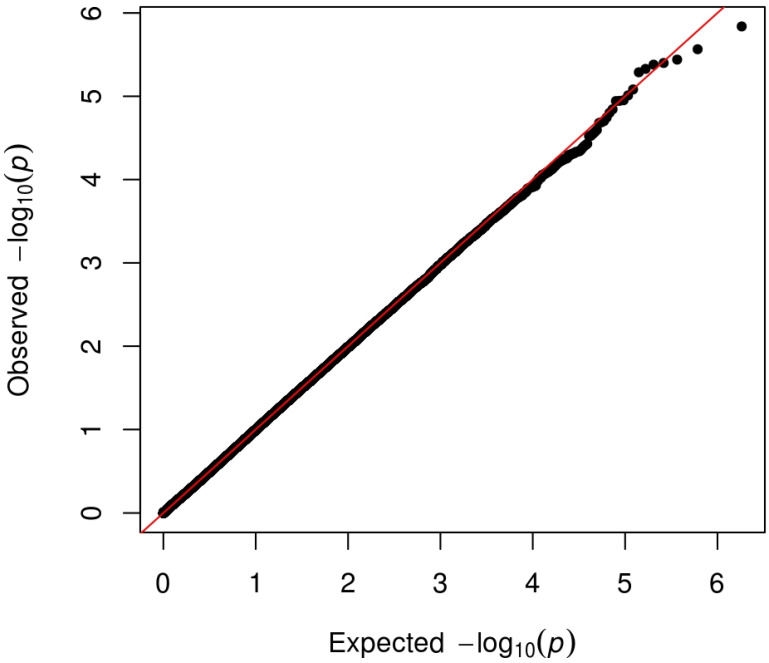
QQ-plot of the TDI association test in the primary sample. The picture shows the QQ-plot analysis. The presence of inflation is excluded from the database under analysis. *x*-axis: Represents the expected *p*-values under the null hypothesis (no significant difference between cases and controls). These values are what you would expect if there were no true associations. *y*-axis: Represents the observed *p*-values from the GWAS analysis, which indicate the significance of the associations found in the data. If the observed *p*-values closely align with the expected *p*-values (i.e., points fall along the diagonal line), it suggests that there is no significant genetic stratification in the sample, supporting the null hypothesis. A deviation from this line, particularly where observed *p*-values are significantly lower (indicating higher significance), suggests the presence of true genetic associations. This could indicate genetic stratification, where the cases and controls differ in ways that affect the results. A large number of observed *p*-values that are more significant than expected could indicate that the sample is genetically stratified. This stratification can lead to inflated type I error rates, meaning that the study may falsely identify associations that do not exist. The presence of a small fraction of observed *p*-values that are significantly lower than expected may indicate true genetic associations, which are of interest for further investigation.

**Figure 2:**
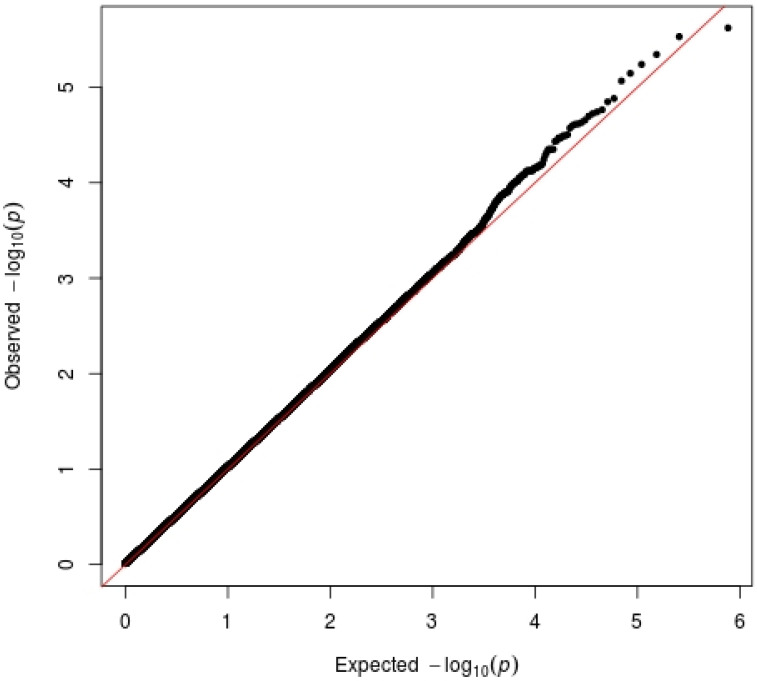
QQ-plot in the secondary sample. The picture shows the QQ-plot analysis. The presence of inflation is excluded from the database under analysis. *x*-axis: Represents the expected *p*-values under the null hypothesis (no significant difference between cases and controls). These values are what you would expect if there were no true associations. *y*-axis: Represents the observed *p*-values from the GWAS analysis, which indicate the significance of the associations found in the data. If the observed *p*-values closely align with the expected *p*-values (i.e., points fall along the diagonal line), it suggests that there is no significant genetic stratification in the sample, supporting the null hypothesis. A deviation from this line, particularly where observed *p*-values are significantly lower (indicating higher significance), suggests the presence of true genetic associations. This could indicate genetic stratification, where the cases and controls differ in ways that affect the results. A large number of observed *p*-values that are more significant than expected could indicate that the sample is genetically stratified. This stratification can lead to inflated type I error rates, meaning that the study may falsely identify associations that do not exist. The presence of a small fraction of observed *p*-values that are significantly lower than expected may indicate true genetic associations, which are of interest for further investigation.

TDI (trait-dependent interaction—this analysis investigates how the interaction between genetics variants can influence the phenotype under analysis) associations were run with Plink, and the results were annotated. The standard quality thresholds were applied to the original sample before pruning and imputing. The minor allele frequency was set at 0.05, the genotype rate was set at 0.95, and the Hardy–Weinberg equilibrium was set at 0.00001. Pruning was set at the standard—indep 50 5 2, where 50 is the number of SNPs considered at every step, 5 is the number of SNPs to be shifted at every step, and 2 is the VIF threshold (1/(1 − R^2^), where R^2^ is the multiple correlation coefficient). A R^2 equal to 10 implies that two SNPs carry the same signal. A R^2^ equal to 1 implies that two SNPs are completely independent. The quality of the results was checked (QQ-plot and λ values; please refer to Figure 1 and Figure 2). QQ-plots and lambda values are essential tools for excluding inflation, which refers to the presence of structural false-positive findings in a dataset. QQ-plots illustrate the deviation from expected to observed significant associations, while a lambda value close to one suggests the absence of inflation in the dataset. Variations associated with autism with a significance threshold inferior to 10 × 10^−4^ were selected and the corresponding genes—genes that harbor the selected variations—identified (genelist1). SNP characteristics and corresponding data are shown in Table 1. Enriched GO (Gene Ontology) terms were searched in genelist1: the GO Consortium served for the analysis (http://geneontology.org/, accessed on 15 July 2024). Such an analysis investigates whether specific terms from the GO database are statistically overrepresented in a given set of genes. Genes within the enriched GO terms were selected (thus, creating genelist2) and their SNPs identified (www.gwascentral.com, accessed on 15 July 2024) through interrogation of the international online databases that pair, when possible, single genetic variations to genes and searched for in the available NIMH GWAS dataset. SNPs belonging to genetlis2 available for the analysis were annotated.

#### 2.3.2. Replication Sample

SNPs were derived from genetlist2, and the incidence of observed vs. expected significant associations was checked in the replication sample.

### 2.4. Selection of Significant Thresholds

Genelist1 was created after selecting SNPs which strength of association with the investigated phenotype was stronger than 10 × 10^−4^. This threshold was chosen in order to prioritize the best available SNPs after the genetic association analysis while retaining a sufficient number of SNPs to identify a list of candidate genes also available in the replication sample. In the following steps of the analysis, the *p* thresholds were set to a less punitive level in order to identify the possible positive associations while abating the risk for false-positive associations with permutations (see Section 2.6). We were interested in analyzing the hypothesis that the prevalence of SNPs from genelist2 associated with the phenotype under analysis was higher—and not lesser—than the prevalence of the same SNPs in genelist2. Since we were not interested in one side of the distribution of probabilities (we were only interested in “more represented” and not “less represented”), a more punitive 0.025 (instead for 0.05) and 0.005 (instead for 0.01) threshold was chosen.

### 2.5. Index Pathway Definition

The index pathway is defined as the molecular pathway that is significantly overrepresented in genelist1. Genes belonging to the overrepresented molecular pathways are stored in genelist2.

### 2.6. Simulation

A subset of SNPs was selected in the replication sample while respecting the following characteristics:

1. should be associated with autism at a *p* threshold < 0.001 in the investigation sample;
2. should be available in the replication sample;
3. should have the same direction of association (OR> or <1 as in the investigation sample).

Permutations were run until a monotone number of different outputs resulted from the process. The permutated molecular pathways had the same length (number of SNPs) of the pathway in list1 in order to avoid an overrepresentation of SNPs associated with the phenotype under analysis due to the difference in lengths of the permutated molecular pathways (more genes equals to higher probability to analyze more SNPs). The probability of having a random pathway presenting with a number of significant (*p* < 0.05) associations superior to or equal to the index pathway (extracted from the investigation sample and tested in the replication sample) was then calculated.

Therefore, 10 × 10^6^ permutations were conducted in the investigation sample, creating a number of molecular pathways similar to the index pathway in terms of the number of missense splicing variations and variations without annotation. The probability of having a random pathway presenting with a number of significant (*p* < 0.05 and *p* < 0.01) associations superior to or equal to the index pathway was then calculated. Permutations were instrumental in accepting or rejecting the hypothesis that a statistically significant enrichment was observed for the same pathway in both the investigation and replication sample.

#### 2.6.1. Acceptance of H0 (Null Hypothesis)

Under the null hypothesis, enrichment of the index pathway is to be considered a false-positive finding.

Acceptance of the null hypothesis was to take place in case of more than 2.5% and 0.05% of the simulated pathways showing the same or an increased number of SNPs significantly (*p* < 0.05 and *p* < 0.01) associated with the phenotype under analysis.

#### 2.6.2. Acceptance of H1 (Enrichment Hypothesis)

Under H1, enrichment of the index pathway is to be considered a true-positive finding.

Acceptance of H1 was to take place in case of less than 2.5% and 0.5% of the simulate pathways showing the same or an increased number of SNPs significantly (*p* < 0.05 and *p* < 0.01) associated with the phenotype under analysis. In case of the acceptance of H1, SNPs in the replication sample that are harbored by genes in genelist2 were identified and their association (*p*-values) and direction of association (OR> or <1) with autism recorded. SNPs and OR were recorded in list1.

## 3. Results

### 3.1. Investigation Sample

The database under analysis was the Autism Dataset 4 (Study 65/TASC GWAS Data) sample. The phenotype under analysis was the “nimh_autism_dataset4_altpheno_plink.txt”, as downloaded from the NIMH genetics dataset. Therefore, 2890 individuals (1824 males, 1069 females) with non-missing phenotypes were found: 936 cases, 1954 controls, and 3 missing. Additionally,1930 founders and 963 non-founders were found, and the total genotyping rate was 0.93. Also, 965 nuclear families were detected, two founder singletons were found, and 963 non-founders with two parents in 963 nuclear families were included in the analysis. Finally, 934 affected offspring trios were identified, and 1,160,305 markers were available for the analysis.

Genomic inflation was excluded (λ = 1.002) in Figure 1.

The analysis of the molecular pathways associated with genetlis1 resulted in the identification of a single molecular pathway showing a significant overrepresentation. The molecular pathway extracted from genelist1 based on its overrepresentation was the central nervous system development pathway (GO:0007417, index pathway). The results are reported in Table 2. Table 3 shows the genes that belong to GO:0007417 and were found to harbor SNPs associated with autism. Additionally, 1124 SNPs belonging to the index pathway were identified and were available for the analysis in the investigation sample.

The prevalence of significant associations within the pathway was 6.4% at a *p* level of 0.05 (28% more than expected) and 1.3% at a *p* level of 0.01 (30% more than expected).

The number of simulated pathways that had the same or an increased number of SNPs significantly associated with the phenotype under investigation in the investigated sample was 2.2% at a *p* level of 0.05. The null hypothesis was then rejected, and the index pathway was deemed significantly enriched after the TDT in the sample of autistic patients.

### 3.2. Replication Sample

The Autism Dataset 3 (GWAS Data on 1264 Non-AGRE Samples) was the replication sample, and 1233 individuals were available from the dataset: 789 males, 444 females, and 588 cases. Additionally, 333 nuclear families and three founder singletons were detected. Also, 579 non-founders with two parents in 321 nuclear families, 14 non-founders without two parents in 9 nuclear families, and 579 affected offspring trios were detected. Finally, 393,763 markers were included in the analysis.

Genomic inflation was excluded (λ = 1.03) in Figure 2.

**Figure 3:**
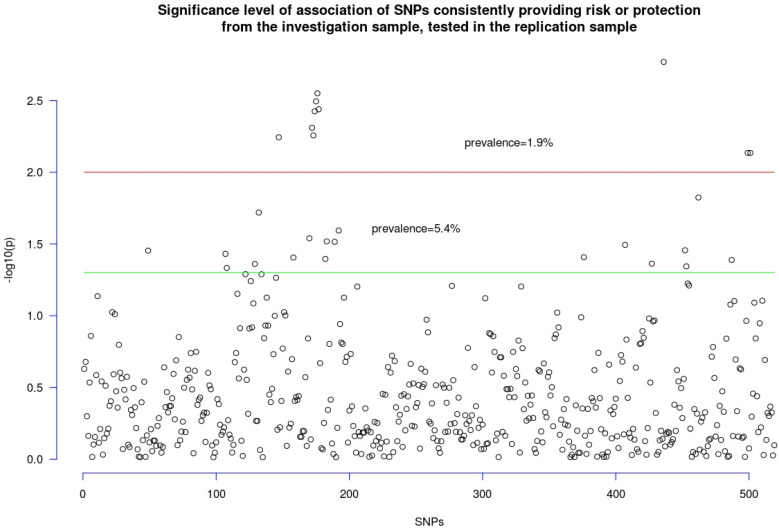
Replication findings. SNPs associated with autism in the investigation sample, available in the replication sample, and having the same direction of association in both samples are shown. The prevalence of SNPs belonging to the index pathway is slightly more superior than expected at a *p* threshold < 0.05 and double the expected at a *p* threshold < 0.01. The latter enriched resisted the permutation test.

The SNPs belonging to the index pathway in the investigation sample (n = 1124) were identified in the replication sample, and 519 SNPs were detected that belonged to the index pathway from the investigation sample and represented in the replication sample and had the same direction of association with autism as in the investigation sample. The prevalence of SNPs associated with autism in the replication sample was 5.4% at a *p* level of 0.05 and 1.9% at a *p* level of 0.01 (Figure 3). After the permutation analysis, the prevalence of SNPs associated with autism was confirmed at a *p* level of <0.01.

As a final result, the hypothesis that the enriched pathway in the investigation sample could be replicated in the replication sample was accepted after the risk for false positives was abated by 10 × 10^5^ permutations. The main results are reported in Table 4 and Table 5.

## 4. Discussion

ASD is a frequent condition in the general population [32] that is characterized by impaired social abilities, restricted interests, and repetitive behavior. The disorder has consistently been shown to have a genetic basis, with the number of involved genes potentially reaching into the hundreds (source: SFARI Gene). This suggests a polygenic nature, indicating that different genetic backgrounds may converge on the same phenotype. To investigate the convergence of specific molecular pathways related to autism spectrum disorder (ASD), a metabolic pathway analysis was conducted using both an investigation sample and a replication sample of autistic trios. The inclusion of a replication sample and the use of permutations were implemented to reduce the risk of false-positive findings. The analysis of complex disorders through a molecular pathway analysis is a well-known and implemented type of analysis, used both in psychiatry and in non-psychiatric disorders [33,34,35]. Research into the genetics of autism has been ongoing for decades, indicating a long-standing interest in understanding its hereditary components. Autism, like many complex psychiatric disorders, is influenced by numerous genetic factors. Individual common genetic variations often have minimal effects on the overall genetic architecture of autism, making it challenging to pinpoint specific risk factors. Genome-wide epigenetic investigations have not yielded conclusive results, suggesting that the interplay between genetics and environmental factors in autism is complex and not fully understood [36]. Increasing sample sizes in genetic studies is a common strategy to enhance the power of detecting associations. Larger samples can help identify variants with small effects that might otherwise go unnoticed. While this approach has led to some interesting findings [37], interpreting these results can be difficult due to the multifactorial nature of autism. A promising strategy to address the limitations of focusing solely on single common variations is the molecular pathway approach. This method combines the effects of multiple genetic variations that contribute to specific biological pathways, potentially providing a more comprehensive understanding of the genetic risk for autism. This approach has shown success in other psychiatric disorders [38,39,40,41], suggesting it could be beneficial for autism research as well. However, its application in autism studies has not been widespread, indicating a gap in the current research methodologies. This finding can be instrumental in developing genetic screening tools for early diagnosis, bridging the gap between psychiatry and other specialties [42,43,44,45]. An interesting approach may be Mendelian randomization, which uses genetic variants that are associated with a modifiable exposure as proxies to infer the causal effect of that exposure on the risk of being diagnosed with autism [46,47].

As a result of this hypothesis-free approach, the GO:0007417 pathway (development of the nervous system) was found to be enriched in the investigation sample. Several variations within this pathway were associated with autism in both samples, as detailed in Table 3 and Table 4. The risk of false positives in the replication analysis was mitigated through permutations, and the findings remained significant at a nominal *p*-value of 0.01, as illustrated in Figure 3. This supports the theory that autism is a neurodevelopmental disorder and is further confirmed by previous studies [48,49].

The present investigation offers a list of SNPs and identifies a GO molecular pathway associated with an increased risk of autism. This identification enhances our understanding of the molecular mechanisms involved in autism, potentially paving the way for new treatments. In this context, we recommend further analysis of the following genes, as reported in Table 5: PCDH9 (Protocadherin 9), also previously found to be associated with autism [50]; TRPC4 (Transient Receptor Potential Cation Channel Subfamily C Member 4), also previously associated with autism [51]; and TMPRSS4 (Transmembrane Serine Protease 4), also previously found to be potentially associated with autism [52].

**Figure 4:**
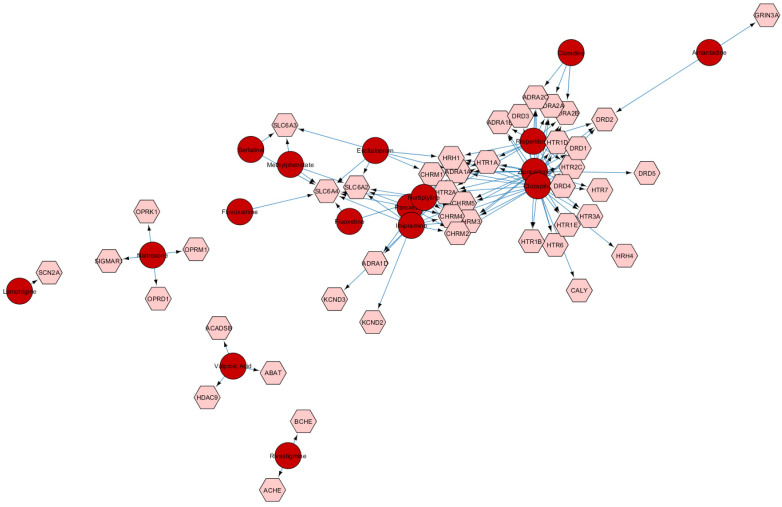
The network below represents the target of molecular drugs used in the treatment of non-core symptoms of autism.

This aspect is of prime relevance, in that most patients with autism are pharmacologically treated in order to target non-core symptoms of their disease, including self-injury, aggression, irritability, and hyperactivity [53]. The molecular pathways that are targets for such treatments are represented in Figure 4. Some drugs have been used in order to treat the target core of the disorder, including growth factors [54], glutamatergic drugs [55,56,57], GABAergic drugs [58,59], and oxytocin [60]. The results are encouraging, but further research is required [61].

The network was built from the Drugbank database. The query was performed through the CyTargetLinker plugin of Cytoscape 3.10.3 software.

Dark-shadowed hexagons indicate the targeted proteins of pharmaceutical compounds. The proteins were obtained from the previous analyses on GO:0007417. Dark-shadowed circles indicate the compounds which interactions with the targeted proteins have been validated in the Drugbank Database.

**Figure 5:**
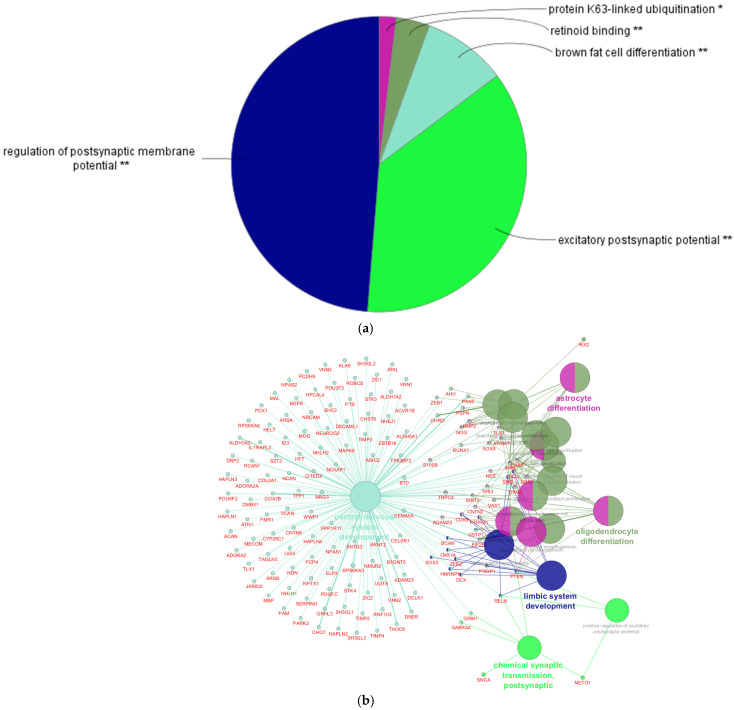
(**a**) In the following diagram, the biologic processes related to the genes under investigation are represented. The diagram shows the subnetworks, and the genes not included in subnetworks, that belong to GO:0007417. In the network below is presented a graphic visualization of the molecular network. The asterisks indicate when the correlation between the subnetworks and the set of genes under investigation is significant (*) or highly significant (**). This was calculated by the Cluepedia plugin for Cytoscape 3.10.3 software. To calculate the degree of significance for each biological process, the software evaluates the ratio between (1) the number of genes within the network under investigation that also belong to the biological process and (2) the total number of genes composing the same biological process. (**b**) GO:0007417. The molecular pathway is represented, along with some of the connected molecular networks, including the astrocyte and oligodendrocyte differentiation, the development of the limbic system, and postsynaptic transmission, among others. The network was built from the GO Biological Process database (9 February 2016). The query was performed through the ClueGO plugin of Cytoscape software. Details on the query, including kappa score, the Tree interval considered, and others, are available upon request.

The GO:0007417 pathway represents a comprehensive network that encompasses several biological subprocesses involved in the development and physiological functions of the central nervous system. A detailed view of the network and its subnetworks within GO:0007417 is illustrated in the figure below (Figure 5a,b). In particular, Figure 5b highlights various molecular interactions associated with GO:0007417, including those related to astrocyte and oligodendrocyte differentiation, the development of the limbic system, and postsynaptic transmission, among others. This representation is particularly noteworthy, as the role of glial cells in shaping neuronal networks during development may be crucial for understanding several psychiatric disorders, including autism [62]. Moreover, the involvement of the limbic system may play a major role in the pathophysiology of autism, which is characterized by a diminished ability to process affective inputs [63,64].

Radial glia cells help neurons migrate towards their final neurological destination through the developmental process, starting from the fetal period [65]. The migration from the germinal areas of the nervous system, which is dependent on the correct activity of the glia system, shapes some nervous structures later during the developing period, called cortical mini columns [66]. Those structures were found to be significantly altered in the brains of autistic patients, containing more neurons when compared to the normal controls and signs for a global GABAergic dysfunction [67]. Finally, the glia is involved in the pruning process, an event that continues in late adolescence and is central to the development of brain structures. This process is mediated in part by glia cells [68], as it was demonstrated that the glia is overrepresented in the brains of autistic patients, which shows a pattern of activation that is aberrant or consistent with a pro-inflammatory state [69].

This is consistent with the hypothesis that autism arises from disruptions in the physiological interregional connectivity between brain regions. Our findings align with previous observations in the literature and suggest an involvement of neurodevelopmental events, as well as a potential role of glial cells in shaping these processes. However, the present investigation has limitations that necessitate further independent studies.

One significant limitation is the low number of markers and the inconsistencies between markers in the investigation and replication samples. This resulted in a heterogeneous representation of the same molecular pathways across the two genetic databases. Replication was possible because different genes covered in the various genetic databases were found to be enriched and clustered within the GO:0007417 pathway. The lack of comprehensive coverage and the differing SNPs included in the two arrays may have contributed to a decrease in the power of the current analysis.

The linkage disequilibrium (LD) structure of the variations under analysis was considered in the quality assessment of the results (using QQ-plots and lambda values), but the impact of LD cannot be entirely ruled out. The samples analyzed are considered medium to large in the context of GWAS, which may also have influenced the power of this investigation. We attempted to mitigate the risk of false-positive findings by including a replication sample and conducting permutation analyses. Nevertheless, further independent analyses are essential before confirming our results.

Finally, our results strengthen the idea that further research is needed focused on the genes and molecular cascades that characterize the GO:0007417 pathway. These genetic insights could inform the development of targeted therapies for autism.

## 5. Dataset

### 5.1. Investigation Sample

Genetic data were available from the NIMH Center for Collaborative Genetic Studies.

NIMH Study 65, also known as AGP or TASC (PI: Gallagher), deposited genotype data in four sets, cleaned and raw, stage1 and stage2. NIMH then combined the cleaned stage1 and stage2 data into one dataset named Autism Dataset 4 in the PLINK file format. The n = 2893 records in Dataset 4 occur in n = 964 families (99% trios), of which n = 935 families have one or more probands with a diagnosis, ranging from strict to broad autism; see the documentation for definitions.

The AGP Simplex Collection (TASC) was funded by an award from Autism Speaks and by funding support to the repository development by the NIMH. The principal investigator and co-investigators in this study were Louise Gallagher, Trinity College Dublin; Astrid Vicente, Instituto Gulbenkian de Ciencia, Oeira; Joseph Buxbaum, Mount Sinai School of Medicine; Peter Szatmari, McMaster University; William McMahon, University of Utah; Michael Cuccaro, University of Miami; James Sutcliffe, Vanderbilt University; Christine Freitag, Klinikum der Johann-Wolfgang Goethe-Universität, Frankfurt/Main; Sabine Klauck, Deutsches Krebsforschungszentrum (DKFZ), Heidelberg; Veronica Vieland (DCC Director), Research Institute at Nationwide Children’s Hospital, Ohio; Dan Geschwind, AGRE/UCLA; John Nurnberger, University of Indiana; Ed Cook, University of Illinois at Chicago; Raphael Bernier, University of Washington/CPEA.

### 5.2. Replication Sample

Genotype data were generated by Dan Arking and Aravinda Chakravarti using 1264 samples that were obtained from the NIMH Center for Collaborative Genetic Studies on Mental Disorders. This study was supported by grants from the NIMH (R01 MH060007) to Aravinda Chakravarti and Dan Arking.

Arking DE, Cutler DJ, Brune CW, Teslovich TM, West K, Ikeda M, Rea A, Guy M, Lin S, Cook EH, Jr., Chakravarti A. A Common Genetic Variant in the Neurexin Superfamily Member *CNTNAP2* Increases Familial Risk of Autism. *Am J Hum Genet* 2008; 82:160–164.

### 5.3. Computational Power

A computer cluster was used to undertake the analyses. Computation power was required for the permutation analysis. The GenomeDK HPC cluster at Aarhus University comprises 190 nodes (3384 compute cores) connected with 10GigE/Infiniband. Each node has from 16 to 32 cores and either 64 GB, 128 GB, 256 GB, 512 GB, or 1 TB of RAM. The cluster has been designed specifically for bioinformatic workloads and has a storage capacity of 3.5 PB.

## Figures and Tables

**Table 1:** SNPs associated with autism with *p* < 10 × 10^−4^.

Gene	Sequence	Position	SNP
CAMTA1[intronic]	>>NNNTTACAGT(C):(T)TTAACCACTG>>	chr1:7,143,344..7,143,344	rs2187901
C1orf110(dist = 3597), RGS4(dist = 196194)[intergenic]	>>aaaataactg(C):(T)aagaaaaaag>>	chr1:162,842,202..162,842,202	rs12047510
LOC642587(dist = 111217), CAMK1G(dist = 39936)[intergenic]	>>aaggtgcaaa(T):(G)atctatgcat>>	chr1:209,717,109..209,717,109	rs4844851
ESRRG[intronic]	>>atgattcccc(C):(T)atcctggtat>>	chr1:216,966,601..216,966,601	rs7355008
C1orf110[intronic]	>>aagaaaaaga(C):(T)atacagactg>>	chr1:162,835,703..162,835,703	rs12137443
C1orf110(dist = 28505), RGS4(dist = 171286)[intergenic]	>>GGATCCAGGG(T):(G)CATAGAACAC>>	chr1:162,867,110..162,867,110	rs7533937
TTC7A[UTR3]	>>CTGTGGTTGC(C):(T)AGATCCAGTC>>	chr2:47,301,744..47,301,744	rs1046263
DKFZp686O1327(dist = 12942), PABPC1P2(dist = 1497392)[intergenic]	>>GAAAAAAAAA(A):(G)TCAATGCCCA>>	chr2:145,847,233..145,847,233	rs2663966
TFPI(dist = 395225), GULP1(dist = 342946)[intergenic]	>>caataagaga(C):(A)atctgatgta>>	chr2:188,814,444..188,814,444	rs10170218
TFPI(dist = 411011), GULP1(dist = 327160)[intergenic]	>>aacaataata(C):(T)aGTTAGAAtg>>	chr2:188,830,230..188,830,230	rs12105426
TMEM194B(dist = 39273), NAB1(dist = 75107)[intergenic]	>>TCATCCAGCT(C):(A)GGAAGCTGCA>>	chr2:191,438,741..191,438,741	rs7603849
TMEM194B(dist = 46800), NAB1(dist = 67580)[intergenic]	>>TACCTTAGTT(T):(G)ATAATGTACA>>	chr2:191,446,268..191,446,268	rs11680758
TMEM194B(dist = 53431), NAB1(dist = 60949)[intergenic]	>>AGACATTTTT(T):(G)CAGTGGGAcc>>	chr2:191,452,899..191,452,899	rs4321393
TMEM194B(dist = 93698), NAB1(dist = 20682)[intergenic]	>>TGCTAGTTAA(A):(G)AGCATGTATA>>	chr2:191,493,166..191,493,166	rs887695
SH3BP4(dist = 245560), AGAP1(dist = 192818)[intergenic]	>>GCATCAATGA(A):(G)CCTCAAGAAA>>	chr2:236,209,918..236,209,918	rs13019316
MECOM(dist = 14218), TERC(dist = 86617)[intergenic]	>>ATGCCTATCT(A):(G)GCCTACGGCA>>	chr3:169,395,781..169,395,781	rs17507954
ZNF385D (dist = 108667), UBE2E2(dist = 1343301)[intergenic]	>>TTTTTAATTT(C):(T)TGATACTTTT>>	chr3:21,901,483..21,901,483	rs1679235
PPP2R2C[intronic]	>>ATTCAGTGTG(T):(G)CTTCATCTCC>>	chr4:6,523,276..6,523,276	rs4075006
GPRIN3(dist = 179798), SNCA(dist = 236291)[intergenic]	>>CTTCGAGATG(A):(G)TTTCTACTTA>>	chr4:90,408,959..90,408,959	rs17015665
GPRIN3(dist = 206392), SNCA(dist = 209697)[intergenic]	>>CGGTCATTAC(T):(G)AGATAGCATT>>	chr4:90,435,553..90,435,553	rs924033
IRX4(dist = 740038), IRX2(dist = 123361)[intergenic]	>>ATGCCTGCTG(C):(T)GGAGGAGGTG>>	chr5:2,622,918..2,622,918	rs172834
LOC100505738(dist = 492614), SEMA5A(dist = 79315)[intergenic]	>>CATAAGAAAA(T):(G)ACCACAAGGA>>	chr5:8,955,823..8,955,823	rs6874770
RAB3C[intronic]	>>CTAACACTGC(T):(G)CTAATTAAGA>>	chr5:57,916,579..57,916,579	rs386161
SLCO4C1[UTR3]	>>CAAGGGCTGC(A):(G)TTGTATTTTA>>	chr5:101,570,236..101,570,236	rs3811976
AKAP7(dist = 34960), ARG1(dist = 254730)[intergenic]	>>TTCTGAGGAC(C):(T)GATCAGAGAT>>	chr6:131,639,635..131,639,635	rs1322595
NXPH1(dist = 200291), PER4(dist = 681016)[intergenic]	>>ATCAGTTGGG(C):(T)CTATCATTAT>>	chr7:8,992,884..8,992,884	rs10250332
C7orf69, PKD1L1[intronic]	>>ATTTGTTTAC(A):(G)TGCATGTCTG>>	chr7:47,835,174..47,835,174	rs4724645
C7orf69, PKD1L1[intronic]	>>tgtaTTCTTT(C):(T)ACTTCAGGAG>>	chr7:47,846,770..47,846,770	rs2348460
STAG3L4(dist = 1500655), AUTS2(dist = 776737)[intergenic]	>>TCTCTGTGAC(A):(G)TTCCTTCCAA>>	chr7:68,287,168..68,287,168	rs6460468
PCLO[intronic]	>>GAATGCATAC(C):(A)AAACAAAATT>>	chr7:82,653,249..82,653,249	rs6977045
PCLO[intronic]	>>TTATACTGCA(A):(G)TATATCCTAA>>	chr7:82,659,856..82,659,856	rs12707538
CDK6(dist = 157200), SAMD9(dist = 105685)[intergenic]	>>AGGCATTTTC(A):(G)GAACCCGCTA>>	chr7:92,623,141..92,623,141	rs1013803
RELN[intronic]	>>CAGGGTTAAA(C):(T)TTAGTACTCA>>	chr7:103,511,617..103,511,617	rs17133124
RELN[intronic]	>>AAACCAATGA(A):(G)ATATAATGCT>>	chr7:103,512,401..103,512,401	rs17133142
DPP6[intronic]	>>TAATACAATC(C):(T)GTTATGTAAA>>	chr7:153,720,890..153,720,890	rs3852281
LZTS1(dist = 462664), LOC286114(dist = 256030)[intergenic]	>>CTCAAACCCA(C):(T)GCAGCTCAAG>>	chr8:20,575,467..20,575,467	rs6984463
CA8[intronic]	>>AATATATAGT(C):(T)TGTTTTATAT>>	chr8:61,120,636..61,120,636	rs13261565
KCNQ3[intronic]	>>TGTCAACACA(C):(T)AAATAAGTGA>>	chr8:133,286,978..133,286,978	rs4736412
COLEC10(dist = 15923), MAL2(dist = 85485)[intergenic]	>>CCTAGCTCTT(C):(T)AGTCTTGTAG>>	chr8:120,135,125..120,135,125	rs13269842
LCN12(dist = 9064), PTGDS(dist = 12943)[intergenic]	>>CTTCCCCGGC(C):(T)GTGTTTGTGT>>	chr9:139,859,013..139,859,013	rs7040970
LCN12(dist = 10315), PTGDS(dist = 11692)[intergenic]	>>GCAGCTGGCC(C):(T)GATGTGAGCC>>	chr9:139,860,264..139,860,264	rs11145951
CCDC3(dist = 87431), OPTN(dist = 10947)[intergenic]	>>ACTACACAGC(A):(G)GACCATATAA>>	chr10:13,131,135..13,131,135	rs11258183
NCRNA00245(dist = 234721), C10orf11(dist = 139058)[intergenic]	>>AGACCTCTTC(C):(T)AACATTAGAA>>	chr10:77,403,461..77,403,461	rs4746315
KCNMA1[intronic]	>>AATCAAGATG(A):(G)TAAGAAAGAT>>	chr10:79,212,639..79,212,639	rs16934809
NRG3[intronic]	>>tctcttctca(A):(G)tttctcaact>>	chr10:83,964,496..83,964,496	rs1764072
PTEN(dist = 71081), RNLS(dist = 234008)[intergenic]	>>GCAAGACCAA(A):(G)TGTGTGTAGG>>	chr10:89,799,613..89,799,613	rs692788
MAML2[intronic]	>>TTATAATTTC(A):(G)TTTATGCCCT>>	chr11:95,991,923..95,991,923	rs1978763
DSCAML1[intronic]	>>TTCCTGGAGC(A):(G)AACTTGGCAG>>	chr11:117,661,100..117,661,100	rs625447
OR8B8(dist = 59751), OR8B12(dist = 41886)[intergenic]	>>AAGCTTTTGA(A):(G)TTTGCCTCAG>>	chr11:124,370,732..124,370,732	rs7119089
OR8B8(dist = 75030), OR8B12(dist = 26607)[intergenic]	>>GAAACTTCTT(C):(T)ATAAATCAAG>>	chr11:124,386,011..124,386,011	rs4282990
FAR1(dist = 199511), SPON1(dist = 30510)[intergenic]	>>AGGATTTCAG(C):(T)CTACAGAGGA>>	chr11:13,953,404..13,953,404	rs16913463
SHANK2[intronic]	>>TCATCCACAG(C):(G)AGGCCCTGCT>>	chr11:70,801,075..70,801,075	rs4627107
SLC16A7(dist = 1133479), FAM19A2(dist = 793154)[intergenic]	>>TTTCTATTAT(A):(G)AAAAATGTTA>>	chr12:61,308,887..61,308,887	rs1480174
TRPC4[UTR5]	>>CTATTTCTTC(A):(G)TCTCTGAAAG>>	chr13:38,357,494..38,357,494	rs12583681
PCDH9[intronic]	>>ATCATCTGAC(T):(G)TAGTTTCACA>>	chr13:67,487,682..67,487,682	rs10492592
CHGA[intronic]	>>CTCAAATGGT(C):(T)CCGCACAGGT>>	chr14:93,400,879..93,400,879	rs750678
CYFIP1[intronic]	>>GGAATTCTGC(C):(T)GTACGTGGTA>>	chr15:22,972,196..22,972,196	rs17841095
LOC145845[ncRNA]	>>AGACAAATTG(A):(G)GACAGGATAT>>	chr15:37,167,090..37,167,090	rs8039031
MEIS2[intronic]	>>CTCTAAATTG(A):(G)CTACAAATTG>>	chr15:37,259,697..37,259,697	rs896052
GCOM1(dist = 25118), ALDH1A2(dist = 210749)[intergenic]	>>ATTTTCAAAC(C):(T)TGTTCCCTTC>>	chr15:58,034,873..58,034,873	rs12912067
FTO[intronic]	>>TTATTGCTAT(C):(T)ACACAGAATT>>	chr16:54,009,328..54,009,328	rs9921255
FTO[intronic]	>>CTGTACTCTA(C):(T)GAATCTTACC>>	chr16:54,009,545..54,009,545	rs9302654
HTA(dist = 218362), LOC100506172(dist = 74670)[intergenic]	>>tgaaattgtg(A):(G)cacgtgacaa>>	chr16:73,346,034..73,346,034	rs6564635
AP2B1[intronic]	>>TAAGCCAAGA(C):(A)AGTGACCGCC>>	chr17:33,988,453..33,988,453	rs9911836
AP2B1[intronic]	>>CTCTTTCCCC(A):(G)ATCTTAAAGA>>	chr17:33,996,764..33,996,764	rs9906872
AP2B1[intronic]	>>CAACAAGGTG(A):(G)GTGTTCAACA>>	chr17:34,042,468..34,042,468	rs9904187
NPTX1[UTR3]	>>GCTGTCAAAG(T):(G)CCCCCAAAAA>>	chr17:78,443,507..78,443,507	rs4889852
NPTX1[exonic]	>>TGGTGGCCCC(T):(G)CCGTAGATCT>>	chr17:78,444,658..78,444,658	rs12943620
None	NA	NA	rs4890026
CBLN2(dist = 158798), NETO1(dist = 39028)[intergenic]	>>accatatcaG(A):(G)TACTTATTTT>>	chr18:70,370,521..70,370,521	rs1564793
BMP2(dist = 950910), HAO1(dist = 151811)[intergenic]	>>AAACCCTGAT(C):(T)TATGATCTAT>>	chr20:7,711,820..7,711,820	rs8121909
XRN2[intronic]	>>TTATATATTA(C):(T)TATTTTTGTC>>	chr20:21,320,653..21,320,653	rs1546995
XRN2[intronic]	>>ATTGTTTTTA(A):(G)TTTAAGCATA>>	chr20:21,324,510..21,324,510	rs6082391
XRN2[intronic]	>>CATTCTTTGA(C):(T)TTCAGATCCA>>	chr20:21,325,711..21,325,711	rs6035850
TMEM189-UBE2V1(dist = 25078), CEBPB(dist = 11963)[intergenic]	>>TGGCACGCCT(A):(G)TAATTAAAGT>>	chr20:48,795,413..48,795,413	rs6125931
TMEM189-UBE2V1(dist = 32659), CEBPB(dist = 4382)[intergenic]	>>TTTCACTACC(A):(G)GTCCTGAGCA>>	chr20:48,802,994..48,802,994	rs6020348
RPS6KA3(dist = 176767), CNKSR2(dist = 931019)[intergenic]	>>GCCAATAGGG(C):(T)CCTTGTTGAG>>	chrX:20,461,517..20,461,517	rs5990877
RPS6KA3(dist = 185595), CNKSR2(dist = 922191)[intergenic]	>>CCTAAGCCTA(C):(T)GGGTGCAATG>>	chrX:20,470,345..20,470,345	rs5950318
RPS6KA3(dist = 197922), CNKSR2(dist = 909864)[intergenic]	>>ATATGGGAGA(C):(T)TTATATACCA>>	chrX:20,482,672..20,482,672	rs10521908
RPS6KA3(dist = 211946), CNKSR2(dist = 895840)[intergenic]	>>TCCCAAGTAG(A):(G)GAATGGGAAG>>	chrX:20,496,696..20,496,696	rs2035111

**Table 2:** Analysis of the overrepresentation of specific molecular pathways.

Upload_1 (*p*-Value)	Upload_1 (Fold Enrichment)	Upload_1 (Over/Under)	Upload_1 (Expected)	Upload_1 (48)	Homo Sapiens—REFLIST (20814)	GO Biological Process Complete
2.84 × 10^−2^	>5	+	2.01	11	870	central nervous system development (GO:0007417)
0.00 × 10^0^	0.81	−	9.85	8	4272	Unclassified (UNCLASSIFIED)

**Table 3:** GO terms enrichment analysis. Genes belonging to the enriched GO pathway are detailed.

Tagging SNPs	Exons	End Position	Start Position	Position	Ensembl	Long Name/Synonyms	Gene
547	9	67369460	66163708	13q21.32	ENSG00000184226	Cadherin (PC00069)	PCDH9
1229	1	117852683	117372348	11q23	ENSG00000177103	immunoglobulin receptor superfamily (PC00197); protein phosphatase (PC00084); protein phosphatase (PC00124); immunoglobulin receptor superfamily (PC00181); immunoglobulin superfamily cell adhesion molecule (PC00195)	DSCAML1
102	11	92871382	92570164	7q21-q22	ENSG00000105810	non-receptor serine/threonine protein kinase (PC00220); non-receptor tyrosine protein kinase (PC00137); non-receptor serine/threonine protein kinase (PC00193); non-receptor tyrosine protein kinase (PC00167)	CDK6
393	11	37905191	37598481	13q13.3	ENSG00000133107	ion channel (PC00227)	TRPC4
203	3	6782147	6765808	replicated_SNPs 20p12	ENSG00000125845	growth factor (PC00207)	BMP2
1664	65	104067175	103394123	7q22	ENSG00000189056	Homo sapiens	RELN
413	17	132534741	132066872	8q24	ENSG00000184156	voltage-gated potassium channel (PC00227); voltage-gated ion channel (PC00133)	KCNQ3
585	22	169750828	168996455	3q26.2	ENSG00000085276	KRAB box transcription factor (PC00218)	MECOM
96	26	20284672	20132332	Xp22.2-p22.1	ENSG00000177189	non-receptor serine/threonine protein kinase (PC00220); transfer/carrier protein (PC00137); non-receptor serine/threonine protein kinase (PC00193); annexin (PC00167); calmodulin(PC00219)	RPS6KA3
225	5	80478069	80465366	17q25.3	ENSG00000171246	antibacterial response protein (PC00090)	NPTX1
78	16	87988203	87847163	10q23.3	ENSG00000171862	protein phosphatase (PC00181); protein phosphatase (PC00195)	PTEN

**Table 4:** SNPs which associations with autism were replicated at a *p* level < 0.01.

Gene	Sequence	Position	SNP
TMPRSS4	>>CTGTCTACCA(C):(T)TTTGGAGCAG>>	chr11:118,002,372..118,002,372	rs11216779
TRPC4	>>atttctagcc(A):(G)ttattattcc>>	chr13:38,777,701..38,777,701	rs2460316
TRPC4	>>gctggcaatg(A):(T)gtcagtacca>>	chr13:38,805,771..38,805,771	rs2202120
TRPC4	>>gaaaatgagt(T):(G)gatacaaaca>>	chr13:38,814,602..38,814,602	rs7320458
TRPC4	>>tggacaagac(A):(G)taaaaggaag>>	chr13:38,814,629..38,814,629	rs7338934
TRPC4	>>gttctgacat(A):(G)tttctagtgt>>	chr13:38,814,687..38,814,687	rs7336867
TRPC4	>>taagtcagaa(C):(T)ttggaactgc>>	chr13:38,814,817..38,814,817	rs6563591
TRPC4	>>TATTCGATTC(A):(G)GAAAAATATG>>	chr13:38,804,435..38,804,435	rs9315565
	>>ACAATTGTAT(A):(G)ACCAGTACAC>>	chr13:68,233,727..68,233,727	rs17619273
PCDH9	>>AAATTCGTCT(C):(G)AAATATAGCA>>	chr13:68,246,757..68,246,757	rs17535905

**Table 5:** Genes which associations with autism were replicated in the present investigation (at least one SNP associated at a *p* level < 0.01).

N Tagging SNPs	N Exons	Stop	Start	Position	Ensembl	Gene
547	9	67369460	66163708	13q21.32	ENSG00000184226	PCDH9
393	11	37905191	37598481	13q13.3	ENSG00000133107	TRPC4
259	16	118132778	118069737	11q23.3	ENSG00000137648	TMPRSS4

## Data Availability

Data are available upon request at https://www.nimhgenetics.org/ (accessed on 15 July 2024).
